# *Aebp2* as an Epigenetic Regulator for Neural Crest Cells

**DOI:** 10.1371/journal.pone.0025174

**Published:** 2011-09-19

**Authors:** Hana Kim, Keunsoo Kang, Muhammad B. Ekram, Tae-Young Roh, Joomyeong Kim

**Affiliations:** 1 Department of Biological Sciences, Louisiana State University, Baton Rouge, Louisiana, United States of America; 2 Department of Biological Sciences, KAIST, Daejeon, South Korea; 3 Division of Molecular and Life Science, Division of Integrative Bioscience and Biotechnology, POSTECH, Pohang, South Korea; Centre National de la Recherche Scientifique, France

## Abstract

*Aebp2* is a potential targeting protein for the mammalian Polycomb Repression Complex 2 (PRC2). We generated a mutant mouse line disrupting the transcription of *Aebp2* to investigate its *in vivo* roles. Aebp2-mutant homozygotes were embryonic lethal while heterozygotes survived to adulthood with fertility. In developing mouse embryos, *Aebp2* is expressed mainly within cells of neural crest origin. In addition, many heterozygotes display a set of phenotypes, enlarged colon and hypopigmentation, similar to those observed in human patients with Hirschsprung's disease and Waardenburg syndrome. These phenotypes are usually caused by the absence of the neural crest-derived ganglia in hindguts and melanocytes. ChIP analyses demonstrated that the majority of the genes involved in the migration and development process of neural crest cells are downstream target genes of AEBP2 and PRC2. Furthermore, expression analyses confirmed that some of these genes are indeed affected in the Aebp2 heterozygotes. Taken together, these results suggest that *Aebp2* may regulate the migration and development of the neural crest cells through the PRC2-mediated epigenetic mechanism.

## Introduction

*Aebp2* is an evolutionarily well conserved *Gli*-type zinc finger gene that is found in species ranging from flying insects to humans [1]. This gene was initially identified due to its binding capability to the promoter of the adipocyte P2 gene, hence named Adipocyte Enhancer Binding Protein 2 (Aebp2) [2]. Since then, *Aebp2* has been increasingly recognized as a component of the mammalian Polycomb Repression Complex 2 (PRC2) due to its frequent co-purification with the other components of PRC2 [3]–[7]. According to recent studies, AEBP2 is indeed a DNA-binding protein with its consensus DNA-binding motif being CTT(N)_15–23_cagGCC. Also, the majority of its genome-wide target sites overlap very well with the known target loci of PRC2, suggesting AEBP2 as a targeting protein for the mammalian PRC2 [1]. Recent studies also provide several mechanisms for PRC2 targeting. *Jarid2* is another gene with potential DNA-binding activity although its binding motifs are very degenerate [3]–[7]. Long non-coding RNAs are also shown to be involved in recruiting PRC2 to a subset of genomic loci. Interestingly, many of these target genes turn out to be cancer-related genes [8]. These studies suggest the presence of many independent targeting mechanisms for PRC2, consistent with the fact that PRC2 likely plays diverse roles in various cell types and tissues [9], [10].

The *in vivo* functions of *Aebp2* are currently unknown, but are likely involved in cell migration based on the following observations. First, *jing*, a *Drosophila* homolog of *Aebp2*, was identified as a gene controlling the border cell migration within eggs [11]. Second, the expression of mouse *Aebp2* is mainly detected within cells of neural crest origin (this study), which are notable for their migratory capability during vertebrate development. Thus, the *in vivo* roles of *Aebp2* are most likely associated with the migration and development of neural crest cells.

The neural crest cell (NCC) is a transient, multipotent cell population that gives rise to many different cell types for vertebrate organs, including those in the enteric nervous system and endocrine system, facial cartilage and bone, and melanocytes. One unique feature associated with NCC is its migration capability from the neural crest to various locations in the developing vertebrate [12], [13]. Several signaling pathways are involved in this migration process, including RET and EDNRB pathways. *RET* encodes a receptor tyrosine kinase that recognizes GDNF (Glial cell line-Derived Neurotrophic Factor) whereas *EDNRB* (Endothelin Receptor B) encodes a G protein-coupled receptor that recognizes EDN3 (Endothelin 3). Mutations in these two pathways quite often manifest as human genetic disorders, including Hirschsprung's disease (HSCR) and Waardenburg syndrome (WS). The disease phenotype of HSCR is obstruction of the gastrointestinal tract, resulting in a pathologically enlarged colon, or ‘megacolon.’ This is caused by the absence of NCC-derived ganglia and subsequent aperistalsis in the colon [14]–[16]. More than half of familial and sporadic cases have been shown to be linked to the *RET* locus although a small fraction of cases are also linked to the EDNRB pathway. On the other hand, the core disease phenotypes of WS are sensorineuronal hearing loss and pigmentary disturbance, which are usually caused by the absence of NCC-derived melanocytes. WS can be further divided into four subgroups based on the presence of additional disease traits: WS Type 1 through 4 [17]–[19]. For example, WS Type 4 (Waardenburg-Shah syndrome) exhibits a similar megacolon phenotype as seen in HSCR in addition to the two WS core traits. WS Type 4 is often caused by mutational defects in several genes in the EDNRB pathway, including *EDNRB*, *EDN3*, and *SOX10* [17]–[19]. Similarly, WS Type 1 through 3 are also linked to the genes encoding transcription factors with significant roles in the migration and development of NCC, such as *PAX3* for WS Type 1and 3, and *MITF* and *SNAI2* for WS Type 2.

In this study, the *in vivo* roles of *Aebp2* have been investigated using a mutant mouse line disrupting its transcription. *Aebp2* is essential for early mouse development based on the lethality observed from Aebp2-mutant homozygotes. During embryogenesis, *Aebp2* is expressed mainly in cells of neural crest origin. Consistently, the heterozygotes display a set of phenotypes that are usually caused by defects in the migration of NCC, suggesting critical roles for *Aebp2* in the migration and development of NCC. The results supporting this conclusion have been presented and discussed in this manuscript.

## Results

### Generation of a mutant mouse line targeting *Aebp2*

**Figure 1:**
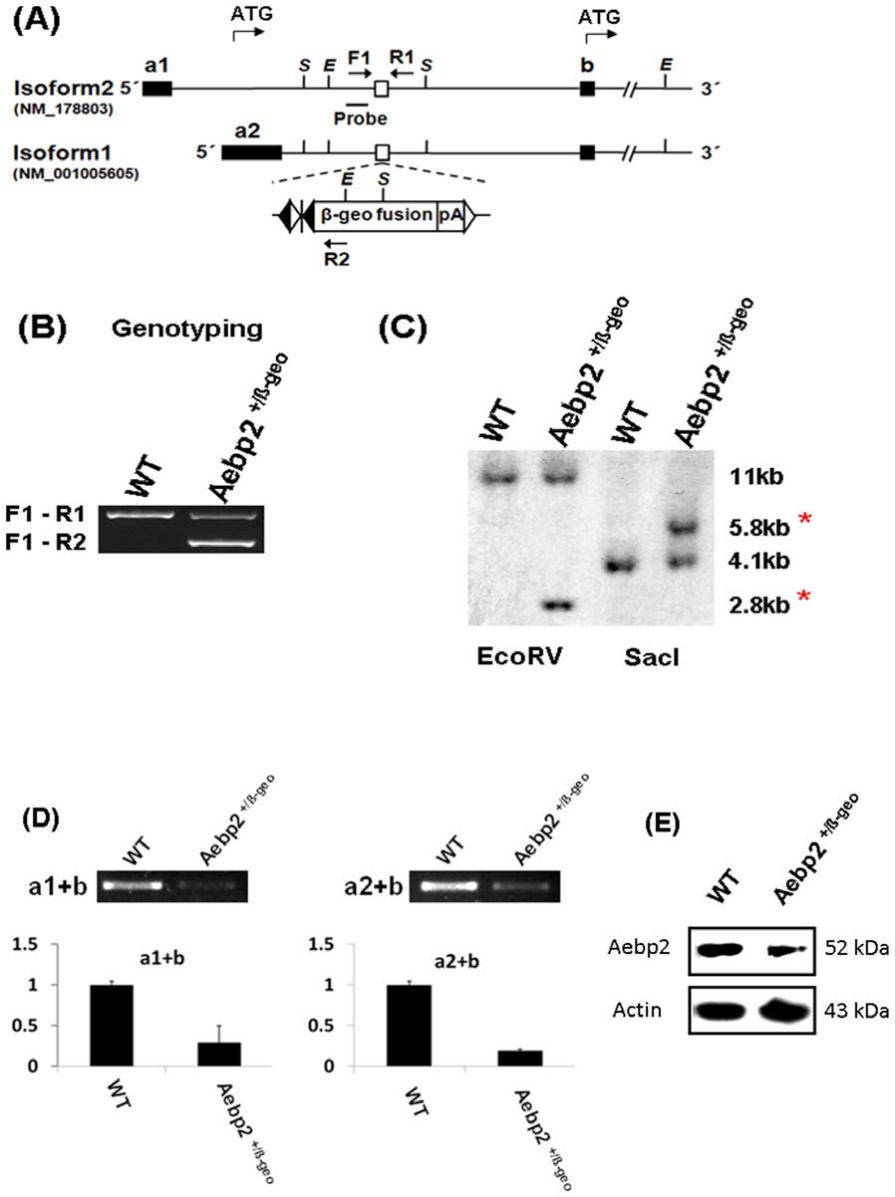
Generation of an Aebp2-knockin allele. (**A**) The gene trap vector has inserted into the first intron of *Aebp2* gene (empty box). This gene trap vector contains two FRT sites (empty triangle), two loxP sites (black triangle, lox71 and loxP), one splicing acceptor site (vertical line), the β-Geo fusion protein cassette, and a polyadenylation signal. Two alternative START codons are indicated with arrow on top. (**B**) Genotyping with three primers (F1, R1, R2). PCR amplification with primers F1 and R1 derives a 570-bp product from the wild-type allele (+), whereas PCR with F1 and R2 produces a 304-bp product from the knockin allele (−). (**C**) Southern blot analysis using genomic DNA (10 µg) from the wild-type (Aebp2^+/+^) and heterozygote (Aebp2^+/β-Geo^) after restriction enzyme digestion with *EcoR*V and *Sac*I. The wild-type and knockin (asterisk) alleles were detected as expected. (**D**) qRT-PCR analyses with the total RNA from the neonatal brains of the wild-type and heterozygotes confirm the proper truncation of the Aebp2 transcripts by the gene trap vector. (**E**) Western blotting using the protein extracts from neonatal brains confirmed reduced levels of the AEBP2 protein in the Aebp2^+/β-Geo^ mice relative to the wild-type littermates. The original image for this western result is available as **Figure S1**.

To characterize the *in vivo* functions of *Aebp2*, we generated a mutant mouse line with one gene trap ES clone (BC0681; http://www.sanger.ac.uk/PostGenomics/genetrap/). After we established this mutant line, we first characterized the insertion position of the gene trap vector (β-Geo). As shown in **Fig. 1A**, the β-Geo vector has inserted into the 1^st^ intron of *Aebp2*. We identified the 5′- and 3′-side junction regions between the β-Geo vector and the surrounding genomic regions, which subsequently allowed us to develop a set of three primers that could be used for genotyping the embryos derived from the breeding of this mutant line (**Fig. 1B**). We also confirmed that the gene trap vector inserted into only the *Aebp2* gene locus with a series of southern blot experiments (**Fig. 1C**). To test the truncation of Aebp2 transcription by the β-Geo vector, we performed qRT-PCR assays using total RNA isolated from the brains of one-day-old neonates [wild-type (Aebp2^+/+^) and heterozygotes (Aebp2^+/β-Geo^)] (**Fig. 1D**). According to separate qRT-PCR measuring the expression levels of two alternative forms, the expression levels of *Aebp2* in the heterozygote were much lower (about 30%) than those detected in the wild-type littermate, confirming the proper truncation of Aebp2 expression by the gene trap vector (β-Geo). We also confirmed this through western blotting (**Fig. 1E**).

### Breeding experiments of the *Aebp2* mutant line

We performed two series of breeding experiments to test potential *Aebp2* roles for the normal development and survival of the mouse. First, we performed the following three breeding experiments: male or female heterozygotes with their littermates and an intercrossing between two heterozygotes (**Table 1**). The results revealed slight reduction in the litter size for both breeding although statistically inconclusive (*p* value being around 0.2): 8 for both F(+/−)×M(+/+) and F(+/+)×M(+/−) vs. 9 for the control breeding F(+/+)×M(+/+). The ratios between the heterozygote and wild type in both breeding were very close to the expected mendelian raio (1∶1). In contrast, the intercrossing between two heterozygotes derived a much smaller litter size (6) than that of the control breeding (9) (*p* value being 0.0022). Also, none of the homozygotes for the Aebp2-mutant allele were found among the offspring derived from 19 litters, confirming the embryonic lethality associated with the *Aebp2* locus. We also performed another series of intercrossing breeding experiments with timed mating, which allowed us to harvest embryos with two different stages: 10.5 and 14.5 dpc, but we did not obtain any homozygotes among the harvested embryos, suggesting that the lethality likely occurs at least before the organogenesis stage (**Table 2**). In sum, these breeding experiments confirm an essential role for *Aebp2* during early mouse development.

**Table 1:** Genotype distribution of the mice from the breeding of the Aebp2 knockin mice.

Genotype	F(+/−)×M(+/−)	F(+/+)×M(+/−)	F(+/−)×M(+/+)	F(+/+)×M(+/+)
**+/+**	35 (30*)	58 (50)	43 (44)	72 (72)
**+/−**	84 (60)	42 (50)	44 (44)	0 (0)
**−/−**	0 (30)	0 (0)	0 (0)	0 (0)
**No. of mice**	119	100	87	72
**Average litter size**	6	8	8	9
***P*** ** value (T-Test)**	0.0022	0.2002	0.2550	#

*This indicates the expected number of F2 pups based on the Mendelian ratio.

# T-Test was perform against the cross between F(+/+)×M(+/+).

**Table 2:** Genotype distribution of the embryos from the breeding of F(+/−)×M(+/−).

Genotype	10.5 dpc	14.5 dpc	Live birth
**+/+**	4	8	30
**+/−**	18	12	73
**−/−**	0	0	0
**Resorbed**	0	2	0
**Total No.**	22	22	103

### Spatial and temporal expression patterns of mouse *Aebp2*

**Figure 2:**
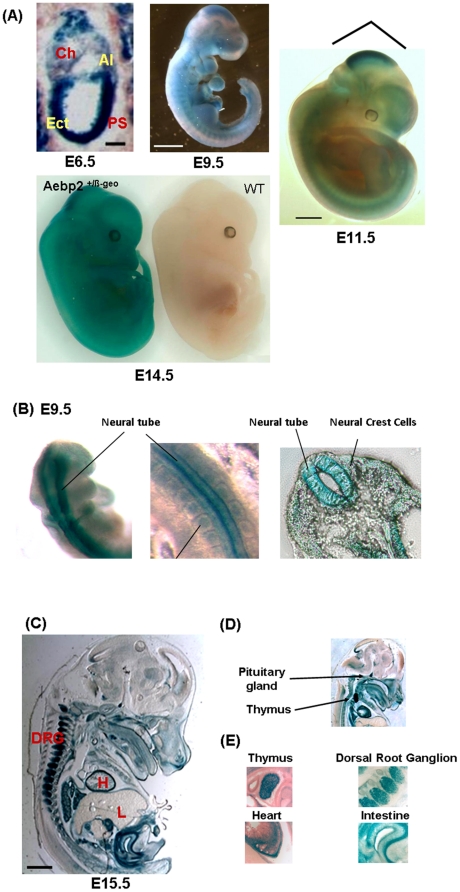
Spatial and temporal expression patterns of *Aebp2*. (**A**) β-Gal staining of whole-mount embryos with different developmental stages. In 6.5-dpc embryos, high levels of *Aebp2* is detected in ectoderm (Ect) and primitive streak (PS), modest levels in chorion (Ch) and allantois (Al). In 9.5-, 11.5-, 14.5-dpc embryos, Aebp2 expression is consistently detected in the midbrain section and also along the somites. Bars, 100 µm (E6.5) and 1 mm (E9.5, 11.5, 15.5). (**B**) β-Gal staining of E 9 embryos: dorsal view showing high expression of *Aebp2* in neural tubes (left) and a zoomed view (center). A transverse section shows *Aebp2* expression in neural tubes and also neural crest cells (right). (**C**) β-Gal staining of a sagittal-sectioned slide from a 15.5-dpc embryo. DRG (Dorsal Root Ganglion), H (Heart), and L (Liver). (**D**) A different sagittal section of a 15.5-dpc embryo showing the expression of *Aebp2* in thymus and pituitary gland. (**E**) Zoom-in views of the Aebp2 expression in the thymus, DRG, intestine, and heart of a 15.5-dpc embryo.

Since the *Aebp2* locus in the mutant line has been targeted by the promoterless gene trap vector (β-Geo), we took advantage of this β-Geo reporter system for analyzing the temporal and spatial expression patterns of mouse *Aebp2*. First, we performed a series of β-Gal staining with whole-mount and cryo-sectioned embryos that had been harvested at various developmental stages (**Fig. 2**). In the sectioned 6.5-dpc embryos, the Aebp2 expression was detected at the highest levels in the embryonic ectoderm (Ect) and primitive streak (PS), and at moderate levels in chorion (Ch) and allantois (Al) (**Fig. 2A**). In the whole-mount embryos with 9.5, 13.5, and 14.5 dpc, the Aebp2 expression was detected in the midbrain section, the branchial arches and along the somites (**Fig. 2A**). This was further confirmed through detecting high levels of Aebp2 expression in neural tubes and neural crest cells in 9.5-dpc embryos (**Fig. 2B**). In the sagittal-sectioned 15.5-dpc embryos, the Aebp2 expression was also detected at relatively high levels in tissues derived from neural crest cells, including dorsal root ganglia, endocrine organs, facial cartilage and bone, and the surface of intestine, heart, and lung (**Fig. 2C–E**). We also performed RNA *in situ* hybridization to confirm independently the initial observation (**Figure S2**), showing no major difference between Aebp2^+/β-Geo^ and wild-type embryos. This further confirms that the observed expression patterns reflect the normal expression patterns of *Aebp2*, but not those of the Aebp2^+/β-Geo^ mice. Second, we also surveyed the sectioned tissues derived from 2-month-old adult mice of both genders. The most obvious expression sites include brain and testes (data not shown). These results are consistent with those from previous studies, revealing high levels of expression in early embryonic stages and adult brains [1], [2]. Overall, it is intriguing that Aebp2 expression is the most obvious in all the tissues derived from the neural crest cell, suggesting significant functional roles for *Aebp2* in the development of this cell lineage.

### Visible phenotypes of the *Aebp2* ^+/β-Geo^ mice

**Figure 3:**
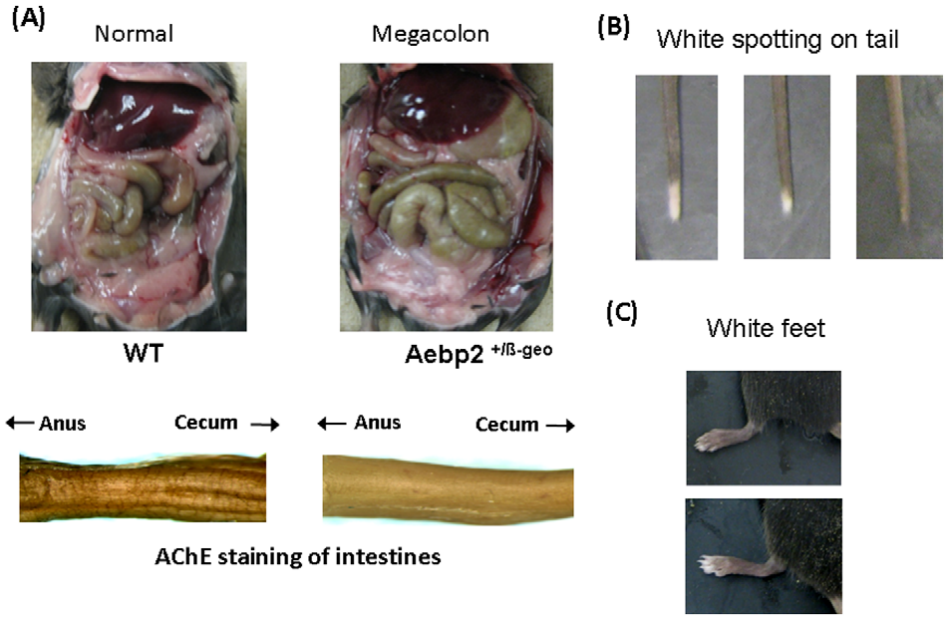
Phenotypes of the *Aebp2* ^+/β-Geo^ mice. (**A**) Comparison of internal organs between the wild-type (WT) and Aebp2^+/β-Geo^ mice (*upper panel*). Some of the Aebp2^+/β-Geo^ mice display an enlarged green-colored colon (Megacolon), which is easily detectible as compared to the normal-size colon from the wild-type mice. Acetylcholinesterase staining further indicates that the Aebp2^+/β-Geo^ mice have much less ganglion cells in the intestinal section between the anus and cecum than the wild-type mice. The ganglion cells are shown as brown thin fibers on the surface of the intestines (*lower panel*). Some of the Aebp2^+/β-Geo^ mice also display white spotting at the tail tip (**B**) as well as the toes (**C**).

While breeding the Aebp2 mutant line, we have observed the following phenotypes from the Aebp2^+/β-Geo^ mice. First, about one quarter of the Aebp2^+/β-Geo^ mice tend to show a pot-shaped belly, and seem to have difficulty in discharging feces. Furthermore, when we examined the internal organs of these mice, some of these mice displayed enlarged, green-colored colons (megacolon, **Fig. 3A**). This megacolon phenotype is caused by the absence of neural crest-derived ganglia and subsequent aperistalsis in the colon [14]–[16]. Thus, the intestines harvested from the Aebp2 heterozygotes were analyzed using the acetylcholine esterase staining method [20], [21]. Out of the 28 Aebp2 heterozygotes examined, 8 mice showed a 50–70% reduced density of ganglion cells in the section between the anus and cecum as compared to the wild-type littermates (**Fig. 3A**).

Second, although we maintained this mutant strain in the 129/B6-mixed background with the black coat color (a/a), we observed 80% of the Aebp2^+/β-Geo^ mice with white spotting at the tail tip (**Fig. 3B**). The length of the white spot area varied among the individual mice of the same litter ranging from 0.2 to 1.5 cm, but the lengths of the white area in the littermates from the intercrossing between the Aebp2^+/β-Geo^ mice were longer than those from the crossing between the wild type and heterozygotes. About 60% of the Aebp2 heterozygotes even showed white toes at the hind limbs (**Fig. 3C**). Third, 70% of the Aebp2^+/β-Geo^ mice did not have a brisk acoustic startle response to clapping sounds, suggesting potential hearing defects, although this needs to be further substantiated through more physiologic and pathologic tests. Overall, the three phenotypes observed from the Aebp2^+/β-Geo^ mice are similar to those observed from Waardenburg syndrome Type 4 (WS4): megacolon, hypopigmentation, and auditory defect.

### *In vivo* binding of AEBP2 and PRC2 to the disease loci of HSCR and WS

**Figure 4:**
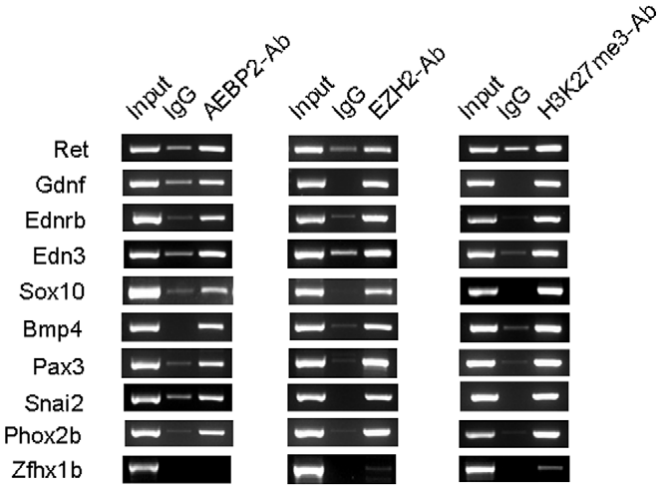
*In vivo* binding of AEBP2 and EZH2 and the methylation level of H3K27me3 on the NCC-associated genes. The cross-linked chromatin from 14.5-dpc embryos was precipitated with the anti-AEBP2, EZH, and H3K27me3 antibodies (left, middle, right). The PCR products from ChIP were presented in the following order: **Input**, **IgG** (pre-immune serum), **AEBP2, EZH2-AB, or H3K27me3**. The majority of the genes involved in the RET and EDNRB pathways, except *Zfhx1b*, are bound by AEBP2 and EZH2, and also modified with H3K27me3.

The HSCR and WS phenotypes observed in the Aebp2 mutant are frequently associated with mutations on a set of about 10 susceptibility genes that are involved in the RET and EDNRB signaling pathways [12], [13]. Since AEBP2 is a DNA-binding protein with NCC-specific expression, *Aebp2* may control these susceptibility loci as a DNA-binding regulator. Therefore, the *in vivo* binding of AEBP2 to the disease loci of HSCR and WS was tested using Chromatin ImmunoPrecipitation (ChIP) experiments (**Fig. 4**). For this series of ChIP experiments, we prepared one set of the cross-linked chromatin isolated from the 14.5-dpc embryos (**Fig. 4**). We selected the promoter region of each of these disease loci for this survey. The majority of these loci except *Zfhx1* were indeed bound by AEBP2 based on the detection of enrichment of the immunoprecipitated DNA by polyclonal AEBP2 antibodies. Since AEBP2 is often co-purified with the mammalian PRC2, we also tested the binding of EZH2 and the methylation on Lys27 of Histone 3 (H3K27me3) to these loci, which represent a key component and a functional outcome of PRC2, respectively. Similar to AEBP2, the majority of the loci except *Zfhx1* also showed the enrichment of the immunoprecipitated DNA by the EZH2 and H3K27me3 antibodies. Overall, the *in vivo* binding of AEBP2 and PRC2 to the disease loci of HSCR and WS suggests that AEBP2 may regulate the disease loci through the PRC2-mediated mechanism.

**Figure 5:**
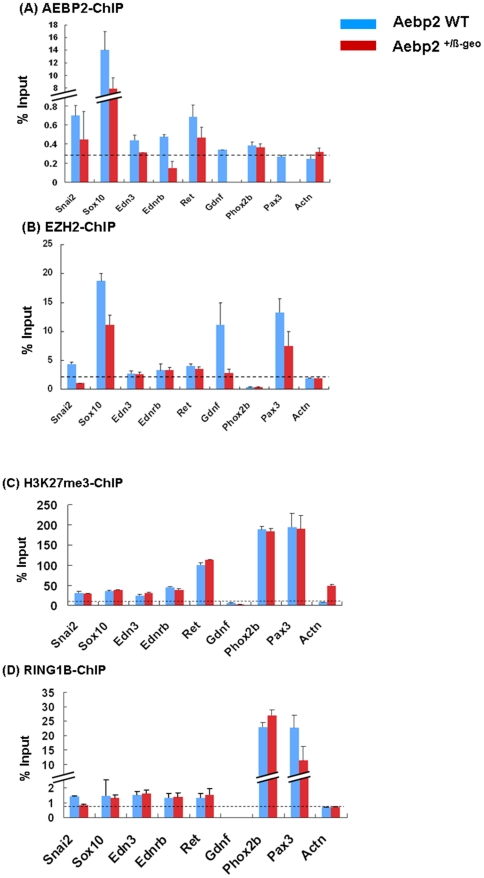
Aebp2 mutation effects on the PRC2-mediated regulation of the NCC-associated genes. The levels of AEBP2 and EZH2-binding to the NCC-associated genes were compared between the wild-type (blue) and Aebp2^+/β-Geo^ (red) embryos with qPCR using the immunoprecipitated DNA derived from 14.5-dpc embryos (**A,B**). The methylation levels of H3K27me3 was also compared between the two types of embryos (**C**). Potential involvement of the PRC1 was also tested using RING1B antibody (**D**). The amount of each precipitated DNA is presented as a relative value (%) to that of the input DNA (y-axis). The values derived from the wild-type and Aebp2^+/β-Geo^ embryos are presented together per each gene (x-axis).

To follow up these initial observations, we performed another series of similar ChIP experiments as described above, and compared the levels of the binding of AEBP2, EZH2, and H3K27me3 to these loci between the wild type and Aebp2^+/β-Geo^ mice (**Fig. 5**). In the majority of the tested loci, the enrichment levels of the precipitated DNA by the AEBP2 antibody were lower in the Aebp2^+/β-Geo^ than in the wild-type embryos (**Fig. 5A**). This is expected since the protein levels of AEBP2 should be lower in the Aebp2^+/β-Geo^ embryos than in the wild-type embryos. This was also the case for EZH2: the enrichment levels on several loci were similarly lower in the Aebp2^+/β-Geo^ embryos, *Snai2*, *Sox10*, *Gdnf*, and *Pax3* (**Fig. 5B**). Interestingly, however, the methylation levels of H3K27me3 on these loci were overall similar between the two groups of embryos (**Fig. 5C**). Although we need to perform more analyses, this might be related to the fact that our ChIP analyses had used the entire body of embryos rather than only the neural crest cells. We also performed another ChIP analyses using the antibody against RING1B, which is a core component of Polycomb Represssion Complex 1 [9], [10] (**Fig. 5D**). The majority of these loci are also bound by RING1B, suggesting potential involvement of the PRC1 in the regulation of these disease loci. Nevertheless, we did not also see any major difference in the enrichment levels by RING1B between the two groups of embryos, which is similar to those observed from H3K27me3. In summary, the similar patterns observed between AEBP2 and EZH2-ChIP further support the initial prediction that *Aebp2* likely controls the genes associated with the migration and development of NCC through the PRC2-mediated mechanism.

### Expression level changes in the disease genes of NCC in the *Aebp2* ^+/β-Geo^ mice

**Figure 6:**
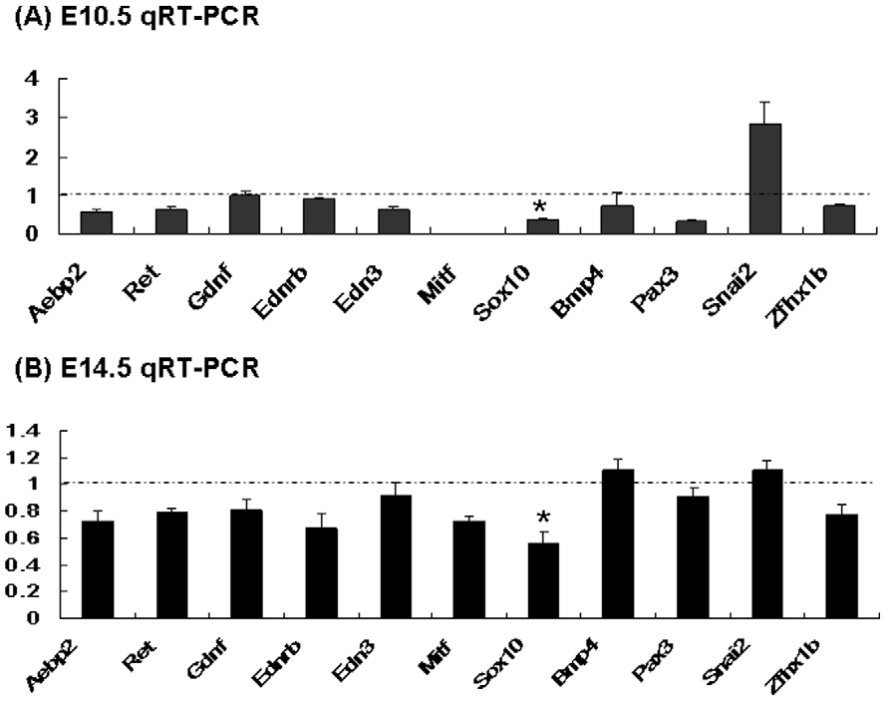
Aebp2 mutation effects on the expression levels of the NCC-associated genes. Expression levels of the NCC-associated genes were compared between the wild-type and Aebp2 heterozygote embryos with qRT-PCR using the total RNA isolated from 10.5 and 14.5-dpc embryos (**A**,**B**). The fold change displayed on each gene indicates its relative expression level in the Aebp2^+/β-Geo^ mice compared to that in the wild-type embryos.

Given the observations described above, it is also likely that the observed phenotypes in the Aebp2^+/β-Geo^ mice may be an outcome of de-regulation of some of the disease loci. To test this prediction, we measured and compared the expression levels of the disease genes between the Aebp2^+/β-Geo^ mice and wild-type littermates (**Fig. 6**). Since the gene dosage (or expression levels) of these loci are critical during embryogenesis, this series of qRT-PCR analyses mainly used the total RNA isolated from the two groups of embryos with three different stages, 10.5, 14.5 and 17.5 (**Fig. 6**). We first calculated the expression level of each gene relative to that of an internal control, β-actin, and later compared these relative values derived from the Aebp2^+/β-Geo^ mice and wild-type littermates. As shown in **Fig. 6**, the expression levels of *Aebp2* in the Aebp2^+/β-Geo^ mice were 0.5 to 0.6-fold compared to those from the wild-type littermates, confirming the disruption of the Aebp2 transcription. In 10.5-dpc embryos, all of the analyzed genes, with the exception of *Mitf*, showed relatively high levels of expression based on their Ct values ranging from 21 through 29 (Ct value of β-actin being 19). Most genes were down-regulated in the Aebp2^+/β-Geo^ mice: the genes with the most significant changes were *Sox10* (0.5 fold) and *Pax3* (0.5 fold). In contrast, *Snai2* showed up-regulation (2 fold), and this up-regulation appears to be very significant based on its high levels of expression (Ct value 21.3). In 14.5-dpc embryos, the majority of the genes in the Aebp2^+/β-Geo^ mice were also down-regulated as seen in the 10.5-dpc embryos. The most significant down-regulation was also observed in *Sox10* (0.5 fold). However, the down-regulation observed in *Pax3* becomes much milder in the 14.5-dpc embryos than in the 10.5-dpc embryos. This is also true for the up-regulation of *Snai2*: 1.1 fold in the 14.5-dpc embryos compared to 2.0 fold in the 10.5-dpc embryos. This trend was also detected in the 17.5-dpc embryos: the majority of the genes displayed very marginal differences in their expression levels between the Aebp2^+/β-Geo^ mice and wild-type littermates (data not shown). Overall, the expression analyses revealed that the majority of the genes involved in the migration and development of NCC are affected during the organogenesis stage (E10.5 to14.5), and that the expression levels of one gene, *Sox10*, is significantly and consistently changed in the Aebp2^+/β-Geo^ mice. This further suggests that the half dosage of *Aebp2* is likely responsible for the phenotypes of HSCR and WS through de-regulation of some of the disease genes of NCC.

## Discussion

In the current study, the *in vivo* roles of *Aebp2* have been investigated using a mutant mouse line disrupting its transcription. *Aebp2* is essential for early mouse development based on the lethality observed from Aebp2-mutant homozygotes (Aebp2^β-Geo/β-Geo^). Furthermore, the half dosage of *Aebp2* appears to be insufficient for the proper development of some neural crest cells that the Aebp2 heterozygotes (Aebp2^+/β-Geo or β-Geo/+^) display a set of phenotypes very similar to those from HSCR and WS. The majority of the genes involved in the RET and EDNRB signaling pathways appear to be downstream target genes of *Aebp2* and PRC2, and also changes in the expression levels of some of these genes are likely accountable for the phenotypes observed in the Aebp2^+/β-Geo^ mice. These results suggest that *Aebp2* may control these genes through the PRC2-mediated epigenetic mechanism, and also that epigenetic mechanisms are likely involved in the pathogenesis of WS and HSCR.

Genetic breeding experiments revealed embryonic lethality in the Aebp2-mutant homozygotes (Aebp2^β-Geo/β-Geo^) but survival of the heterozygotes (Aebp2^+/β-Geo or β-Geo/+^) to adulthood with fertility (**Table 1**). The embryonic lethality of the Aebp2 homozygotes is similar to that observed from the other components of PRC2, such as *Ezh2*, *Eed*, and *Suz12* [22]–[24]. The null mutants for these genes fail to form the three germ layers after implantation, suggesting essential roles for these genes in the lineage specification of the germ layers. Given the interactions between *Aebp2* and PRC2 [9], [10], we predict that *Aebp2* might also play critical roles in establishing the three germ layers. The evolutionary conservation of *Aebp2* is also noteworthy: its homologues are present in species ranging from flying insects to humans [1]. Given this evolutionary conservation, *Aebp2* is most likely involved in the regulation of a large number of genes and pathways, and thus its depletion should be detrimental for the survival of the embryos. Overall, the embryonic lethality observed from the Aebp2-null mutants suggests an essential role for this PcG gene during early embryogenesis.

The expression patterns of *Aebp2* are considered to be ubiquitous, temporally and spatially, according to the results derived from the previous studies [1], [2]. However, one unique observation from this study is the detection of very high levels of Aebp2 expression in neural crest cells during embryogenesis (**Fig. 2**). This unexpected observation appears to be somewhat consistent with Aebp2's functional connection with PRC2. The migratory NCC is regarded as a multipotent stem cell since it gives rise to so many different cell types in the major organs of adult vertebrates [12], [13], [25]. Stem cells are characterized by two core features, multipotency and self-renewal without differentiation, and these features are usually maintained by epigenetic mechanisms, especially by PRC2 [26]–[29]. Migratory NCC likely employs PRC2 to maintain these properties during embryonic development. Therefore, Aebp2 expression in NCC may be designed to provide these two properties to this stem cell population. If this is the case, the other components of PRC2 should also be highly expressed in NCC, as is *Aebp2*. This will require further study in the near future.

Although the homozygotes for the Aebp2-knockin allele are lethal, the heterozygotes are viable and fertile, and display an intriguing set of phenotypes, enlarged colon and hypopigmentation (**Fig. 3**). Since the Aebp2-knockin allele disrupts the transcription of *Aebp2*, this mutation is regarded as a loss-of-function-type mutation. The phenotypes generated by this Aebp2 mutation are also regarded as dominant traits based on their detection in heterozygotes. Therefore, the dominance of these phenotypes is likely an outcome of haploinsufficiency, meaning the reduced dosage of *Aebp2* is responsible for the observed phenotypes. Similar situations also occur in human patients with Hirschsprung's disease (HSCR) and Waardenburg Syndrome (WS). In most cases of these disorders, mutational defects are found in the genes involved in the migration process of NCC, RET and EDNRB signaling pathways [15], [16]. The disease alleles are also loss-of-function-type mutations, and inherited as autosomal dominant traits. Therefore, haploinsufficiency is also the primary mode for the dominant phenotypes by these disease alleles. Overall, there are many similarities between the Aebp2-knockin allele and the disease alleles of HSCR and WS. In particular, the similar mode of the phenotype dominance, haploinsufficiency, may indicate that the migration process of NCC is very susceptible to changes in the gene dosage of the participating loci. Thus, it is likely that the gene dosage of *Aebp2* is very critical for the proper migration and development of NCC.

As a DNA-binding protein, AEBP2 most likely exerts its *in vivo* roles through its unknown downstream genes. As predicted, ChIP experiments confirmed that AEBP2 indeed binds to the majority of the genes involved in the development and migration of NCC during embryogenesis (**Fig. 4**). The AEBP2 binding to these genes also coincides with the binding of PRC2, suggesting potential involvement of PRC2 in the development of NCC. Expression analyses further confirmed changes in the expression levels of some of these genes by the half dosage of *Aebp2* (**Fig. 6**). In particular, one gene (*Sox10*) is consistently down-regulated in the Aebp2^+/β-Geo^ mice. This is analogous to the reduced gene dosage of *SOX10* frequently linked to WS Type 4 in humans. Also, the phenotypes observed in the Aebp2^+/β-Geo^ mice are seen in human patients with WS Type 4 [19]. It is possible that *Aebp2* is responsible for the observed phenotypes via *Sox10*. However, we cannot rule out the possibility that the effects of the Aebp2 mutation might occur more globally and at much earlier stages than described. If this is the case, the observed phenotypes should not be accounted for by the mis-expression of a single gene. This is evidenced by the observation that other genes involved in the migration of NCC are also affected in the Aebp2^+/β-Geo^ mice. It is important to note that the predicted outcome by the half dosage of *Aebp2* is up-regulation of the majority of the NCC genes given the fact that the PRC2 is a repressive complex. However, the majority of NCC genes are down-regulated in the Aebp2^+/β-Geo^ mice, further suggesting that the effects of the Aebp2 mutation might occur at much earlier stages and also more globally. At the same time, it is prudent to note that our experiments have used whole embryos rather than just NCCs (**Fig. 6**), and thus there are some limitations in deriving meaningful conclusions regarding this issue at the moment. Nevertheless, it will be very interesting to determine if the changes in the Sox10 expression are primarily responsible for the phenotypes observed in the Aebp2^+/β-Geo^ mice.

HSCR and WS demonstrate incomplete penetrance mainly due to their oligogenic nature and other non-genetic factors involved in their pathogenesis [14]–[16], [30]. Identification of *Aebp2* as a potential disease locus for these disorders is an intriguing possibility since Aebp2 involvement in these diseases might be through the PRC2-mediated epigenetic mechanism (**Fig. 4**). If *Aebp2* indeed exerts its roles through PRC2, it may require optimal concentrations of the cellular enzymes and substrates necessary for histone modification reactions. The outcome of these reactions may vary depending on the nutritional status and environmental conditions of developing embryos, resulting in different levels of histone modification among individuals. This type of inter-individual differences, also known as epigenetic variations, may be a major factor contributing to phenotypic variations (e.g. incomplete penetrance) [31], [32]. Unfortunately, epigenetic variations have not been discernible by traditional genetic studies, which rely on genetic variations. We predict that this is the case for both HSCR and WS since the majority of the associated disease genes are modified by PRC2 (**Fig. 4**). It is possible that different levels of histone modifications on the disease alleles are accountable for the phenotypic variations (incomplete penetrance) observed for HSCR and WS. In sum, characterizing *Aebp2* as an epigenetic regulator may provide a new and exciting direction for the study of HSCR, WS, and other related disorders.

## Materials and Methods

### Generation and breeding of the *Aebp2* knockin mutant mice

One gene trap clone, BC0681 (strain 129/OlaHsd) from SIGTR (Sanger Institute Gene Trap Resource, http://www.sanger.ac.uk/PostGenomics/genetrap/), was injected into mouse blastocysts to generate chimeric mice. Injection of these cells into C57BL/6 blastocysts was performed at The Darwin Transgenic Mouse Core Facility (Baylor College of Medicine, Houston, TX, USA). The male chimeric mice were bred with female C57BL/6 mice, and the following F1 offspring with agouti coat color was further genotyped to confirm the germiline transmission of the Aebp2-knockin allele. This initial genotyping was performed with PCR using a primer set targeting the NeoR coding region of the gene trap vector (pGT2lxr). All the experiments related to mice were performed in accordance with National Institutes of Health guidelines for care and use of animals, and also approved by the Louisiana State University Institutional Animal Care and Use Committee (IACUC), protocol #10-071.

### Southern blot and genotyping by PCR

Genomic DNA was purified from the spleens of the wild-type and Aebp2^+/β-Geo^ mice with DNAzol (Invitrogen). Ten µg of these genomic DNA was used for each of *EcoR*V and *Sac*I digestion reactions, separated on a 0.8% agarose gel, and finally transferred onto Hybond nylon membranes (Amersham) by capillary blotting. Membranes were hybridized with a ^32^P-labeled probe corresponding the 1^st^ intron region of *Aebp2* (**Fig. 1**).

Mice were genotyped by PCR using the following three primers: F1, 5-ACCAGGGTTGAAACAGAAGAACTCTG-3; R1, 5-AGGTGCTGCACTCACACTCCCA-3; R2, 5-AACGGTAGGATCCCAAGGGCAGTA-3. The 570-bp product by F1 and R1 primers is amplified from the endogenous allele of *Aebp2*, thus representing the wild-type allele. In contrast, since the R2 primer is derived from the gene trap vector, the 304-bp product by F1 and R2 represents the Aebp2 knockin allele. PCR conditions were 33 cycles at 95°C for 30 seconds, 60°C for 30 seconds, and 72°C for 30 seconds. Also, the genders of neonatal mice and embryos were determined by PCR using the primer set of the mouse *Sry* gene under the same PCR conditions described above; mSry-F (5-GTCCCGTGGTGAGAGGCACAAG-3) and mSry-R (5-GCAGCTCTACTCCAGTCTTGCC-3). To prepare genomic DNA from clipped tails or ears, each tissue was incubated overnight at 55°C in the lysis buffer (0.1 M Tris-Cl, 5 mM EDTA, 0.2% SDS, 0.2 M NaCl, pH 8.0, 20 µg/ml Proteinase K). One µl of the lysed extract was first diluted with 30 µl of TE, and one µl of the diluted extract was finally used for each PCR amplification.

### β-galactosidase staining

Pregnant dams with timed mating were sacrificed at various stages during embryonic development. The embryos were fixed overnight in fixing solution (0.2% paraformaldehyde, 0.1 M PIPES buffer pH 6.9, 2 mM MgCl2, 5 mM EGTA). The fixed embryos were then cryo-protected in the PBS buffer containing 30% sucrose and 2 mM MgCl2 at 4°C overnight, or until the embryos sank to the bottom. These embryos were further embedded in OCT and frozen at −80°C. The embedded embryos were sectioned on a crytome (Leica CM1850) to 50 micron thickness and placed onto poly-L-lysine coated slides. The sections were further immobilized in the fixing solution for 10 minutes. After rinsing in PBS for 10 minutes, they were placed in detergent rinse solution for 10 minutes. The sections were then placed at 37°C overnight in the staining solution containing 1 mg/ml of bromo-chloro-indolyl-galactopyranoside (X-gal). For better contrast, the heart and thymus tissue sections were counterstained with eosin Y [33].

For whole-mount staining, embryos were fixed in 4% paraformaldehyde for 2 hours and stained overnight at 37°C in the staining solution containing 1 mg/ml of X-gal. Tissue sections and whole-mount embryos were visualized using a dissecting light microscope (Leica MZ75). Images were captured with a digital camera (Model #4.2 Color Mosaic, Diagnostic Instruments Inc.).

### Acetylcholinesterase Staining

The intestines from one-month old mice were harvested and fixed in 4% paraformaldhyde for 1 hour at 4°C. After incubation in saturated sodium sulfate overnight at 4°C, the intestines were further incubated for 4 hours in the staining buffer (0.2 mM ethopropazine HCl, 4 mM acetylthiocholine iodide, 10 mM glycine, 2 mM cupric sulfate, and 65 mM sodium acetate pH 5.5). Lastly, the acetylcholinesterase activity was detected by incubating the intestines in 1.25% sodium sulfide pH 6 for 1.5 minutes.

### Chromatin ImmunoPrecipitation (ChIP) experiments

Chromatin immunoprecipitations were performed according to the protocol provided by Upstate Biotechnology (Upstate Biotech.) with some modification as described previously [34]. Briefly, mouse embryos at various stages were harvested and homogenized in 10 ml PBS. The samples were treated with formaldehyde to the final concentration of 1% and incubated at 37°C for 10 minutes. Treated samples were sheared by sonication and immunoprecipiated with anti-AEBP2 (Cat. No. 11232-2-AP, ProteinTech Group), EZH2 (Cat. No. ab3748, Abcam), RING1B (Cat. No. ab3832, Abcam), and H3K27me3 (Cat. No. 07-449, Upstate Biotech.) antibodies. Precipitated DNA and protein complexes were reverse cross-linked and purified through phenol/chloroform extraction. Purified DNA was used as template DNA for PCR amplification. PCR reactions were carried out for 40 cycles using standard PCR conditions. The resulting PCR products were run on 1.6% agarose gels containing ethidium bromide. All ChIP assays were performed independently at least three times. The oligonucleotide sequences used for this study are available upon request (or **Material S1**).

### Quantitative reverse transcription PCR and data analysis

Total RNA was extracted from tissues using Trizol (Invitrogen). Reverse transcription was performed using the M-MLV kit (Invitrogen). Quantitative real time PCR was performed with the iQ SYBR green supermix (Thermo Scientific) using the icycler iQ multicolor real-time detection system (Bio-Rad). All qRT-PCRs were carried out for 40 cycles under the standard PCR conditions. We analyzed the results of qRT-PCR based on the threshold (Ct) value. A Δ Ct was first calculated through subtracting the average Ct value of a given target gene from the average Ct value of an internal control (β-actin). Later, the Δ Δ Ct was calculated through subtracting the Δ Ct value of the target gene in the Aebp2 heterozygote from the Δ Ct value of the same gene in the wild-type littermate. Fold differences were determined by raising 2 to the Δ Δ Ct powers [35]. Information regarding individual primer sequences and PCR conditions is available upon request (or **Material S1**).

## Supporting Information

Material S1 **Sequence information for oligonucleotides used for ChIP and RT-PCR analyses.**
(DOC)

Figure S1 **Western blot results of AEBP2 and Actin between the wild-type and Aebp2^+/β-geo^.** The images on left were presented Fig. 1E, which were extracted from the original images on right.
(TIF)

Figure S2 **RNA** ***in situ*** **hybridizations were performed using the two types of embryos.** As shown above, we did not see any major difference between these two groups. This confirms that the half dosage of *Aebp2* most likely has no effect on the expression patterns of *Aebp2* during embryogenesis. Such that, the expression profiles observed through the β-Gal staining should reflect the normal expression patterns of *Aebp2*. *In situ* hybridization was performed as described by Zakin et al. (Zakin L et al. Dev Biol. 2008 323:6–18.) with additional RNase A treatment after hybridization reaction to reduce nonspecific background staining.
(TIF)
