# Supplementary material for: Aebp2 as an Epigenetic Regulator for Neural Crest Cells
Source: PLoS One. 2011 Sep 19;6(9):e25174. doi: 10.1371/journal.pone.0025174 (PMC3176318; doi:10.1371/journal.pone.0025174)
Supplement: Material S1 — Sequence information for oligonucleotides used for ChIP and RT-PCR analyses. (DOC) [file pone.0025174.s001.doc]

**Primer sequences for ChIP and RT-PCR analyses**

>PAX3-A

CTCGGTGTCACGACGGGAGGAGA

>PAX3-B

CTCCGGATCTCGGAGAGCTCCT

>PAX3-RT-A

GCAGAATTACCCACGCAGCGGCT

>PAX3-RT-B

CTCCTGGTACCTGCACAGGATCT

>SOX10-A

GGCCTGAGGCTCAGGGCCCCCCAG

>SOX10-B

CCCCTCGTCTGCTCTGACAGCCT

>SOX10-RT-A

GAGCTCAGCAAGACACTAGGCAAG

>SOX10-RT-B

GAGGGGTGCTCTGGGTTCCCATCTG

>EDNRB-A

GCTTGCTTCTTCCCGGCACATAC

>EDNRB-B

CATGTTACAGCTTGCTCCTGTGA

>EDNRB-RT-A

AGAGGACTGGCCATTTGGAGCT

>EDNRB-RT-B

TGATGGCTAGCGGCAAGCAGAAG

>EDN3-A

GCCTCAGCCAAGTAACTCTGAG

>EDN3-B

ATCAGCTTCCCCGCAACACAGA

>EDN3-RT-A

GCCGCTGCACGTGCTTCACTTAC

>EDN3-RT-B

CTGGTGACATCTCTGGTGCGTG

>RET-A

CGCCTCTAACCCCAGAAGAAG

>RET-B

GCCGGCATAGCCACTTTCTCA

>RET-RT-A

GGCACACCTCTGCTCTATGTC

>RET-RT-B

CTCTGGGATGCAGAGATCCTG

>GDNF-A

CGGCTGCTCAGACTTAGTCTTCT

>GDNF-B

TCCGTAGACCCCCAGTTGGACA

>GDNF-RT-A

GCCGGTAAGAGGCTTCTCGAAG

>GDNF-RT-B

GGCAGCTGCAGCCTGCCGATTC

>BMP4-C

CATCCCAGGGACCAGTGAGAG

>BMP4-D

TCTGGTGGAGGTGAGTCACCT

>BMP4-RT-C

CATTCCGTAGTGCCATTCGGA

>BMP4-RT-D

CTCTCACTGGTCCCTGGGATG

>MITF-A

GGGTTCTGGTCCAAGTCCCAAG

>MITF-B

GAGCTATCCAGACTGACTGTTC

>MITF-RT-A

GGGAACCATTCTCAAGGCCTCTG

>MITF-RT-B

AGGTTGTTGGTAAAGGTGATGGT
