# Supplementary figures and images for: Aebp2 as an Epigenetic Regulator for Neural Crest Cells
Source: PLoS One. 2011 Sep 19;6(9):e25174. doi: 10.1371/journal.pone.0025174 (PMC3176318; doi:10.1371/journal.pone.0025174)

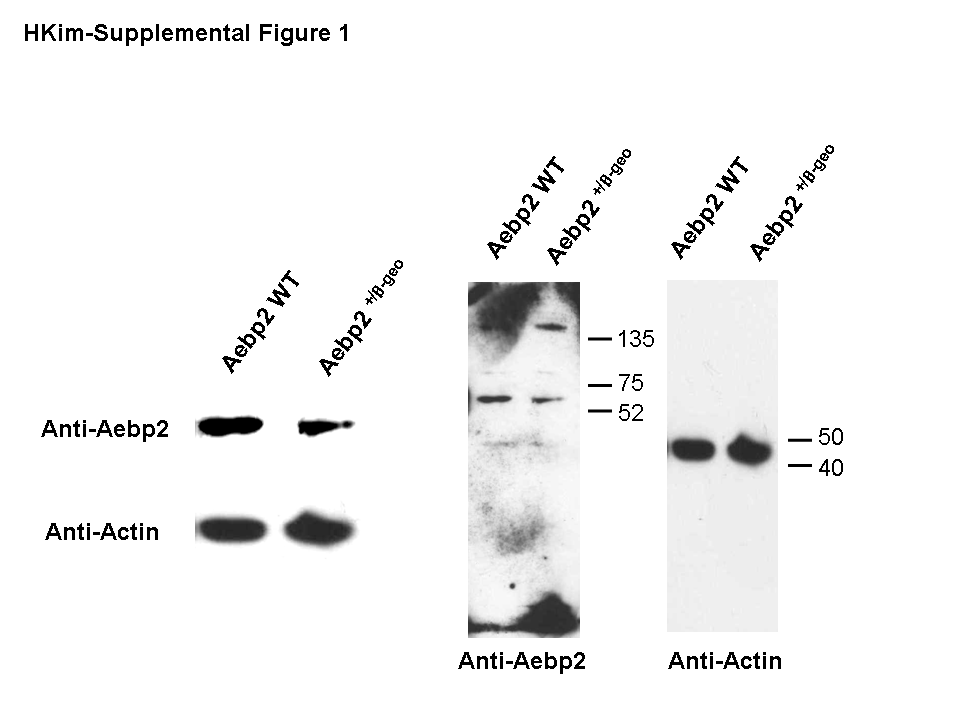

Supplement: Figure S1 — Western blot results of AEBP2 and Actin between the wild-type and Aebp2+/β-geo. The images on left were presented Fig. 1E, which were extracted from the original images on right. (TIF) [file pone.0025174.s002.tif]

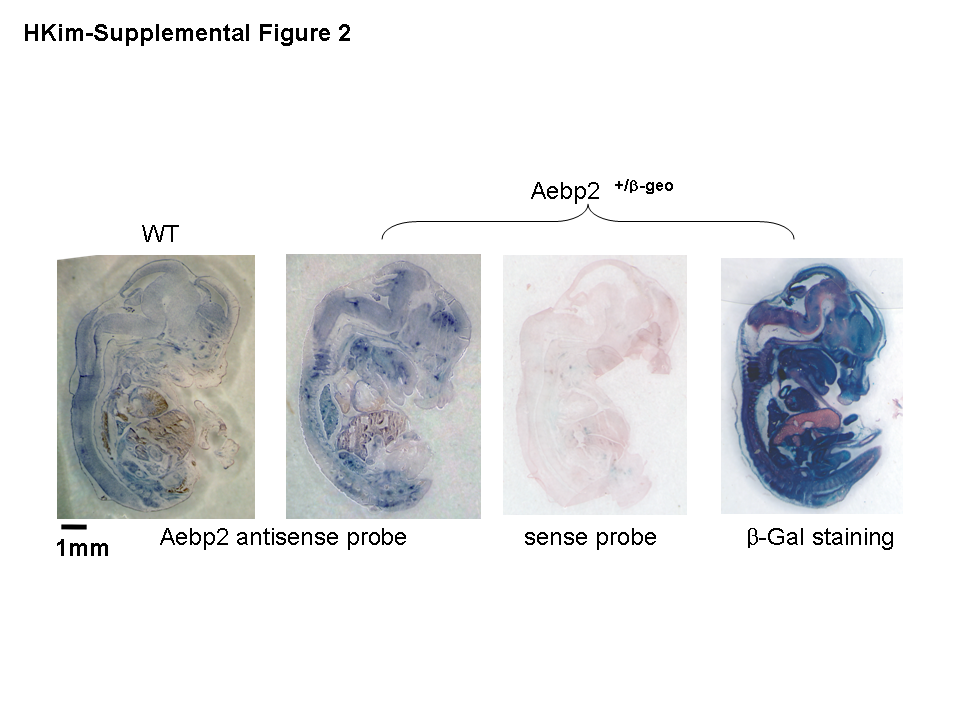

Supplement: Figure S2 — RNA in situ hybridizations were performed using the two types of embryos. As shown above, we did not see any major difference between these two groups. This confirms that the half dosage of Aebp2 most likely has no effect on the expression patterns of Aebp2 during embryogenesis. Such that, the expression profiles observed through the β-Gal staining should reflect the normal expression patterns of Aebp2. In situ hybridization was performed as described by Zakin et al. (Zakin L et al. Dev Biol. 2008 323:6–18.) with additional RNase A treatment after hybridization reaction to reduce nonspecific background staining. (TIF) [file pone.0025174.s003.tif]
